# The WISH 2.0 Intervention for Irritable Bowel Syndrome: Protocol for a Pilot Randomized Controlled Trial

**DOI:** 10.2196/98352

**Published:** 2026-07-23

**Authors:** Elizabeth N Madva, Jeff C Huffman, Helen Burton-Murray, Emmett McGranaghan, Megan Bidgood, Jenny Y Gan, Benjamin Wasserman, Emily H Feig, Brian Healy, Kyle Staller, Bonney Reed, Roberta Sclocco, Sami S Amr, Stephen Bartels, Braden Kuo, Laurie Keefer, Christopher M Celano

**Affiliations:** 1 Harvard Medical School, Mass General Brigham, 55 Fruit Street, Boston, MA, 02114, United States, 1-617-726-2000; 2 Department of Biostatistics, Harvard T.H. Chan School of Public Health, Boston, MA, United States; 3 Division of Behavioral & Mental Health, Emory + Children's Pediatric Institute, Atlanta, GA, United States; 4 Department of Physical Medicine & Rehabilitation, Harvard Medical School, Spaulding Rehabilitation Hospital, Boston, MA, United States; 5 Department of Pathology, Harvard Medical School, Mass General Brigham, Boston, MA, United States; 6 Division of Digestive and Liver Diseases, Columbia University, NewYork–Presbyterian Hospital, New York, NY, United States; 7 Gastroenterology and Psychiatry, Icahn School of Medicine at Mount Sinai, New York, NY, United States

**Keywords:** irritable bowel syndrome, functional gastrointestinal disorders, disorders of gut-brain interaction, positive psychology, psychological resilience

## Abstract

**Background:**

There is a critical need for acceptable, scalable, and mechanistically targeted behavioral health interventions for irritable bowel syndrome (IBS), known as brain-gut behavior therapies (BGBTs). Although positive psychology (PP) interventions, which focus on the cultivation of positive psychological well-being, have been linked to improved health outcomes across diverse medical populations, they have not yet been investigated in IBS. To address this gap, we developed WISH (Well-Being in IBS: Strengths and Happiness), a novel, 9-week, phone-delivered PP-based BGBT for individuals with IBS.

**Objective:**

This randomized controlled trial (RCT) aims to evaluate the feasibility, acceptability, preliminary effects, and candidate gut-brain mechanisms of a 9-week phone-delivered PP intervention for IBS compared with a time- and attention-matched educational control.

**Methods:**

The WISH 2.0 trial is a parallel-group, 9-week pilot RCT that aims to enroll 50 adults meeting Rome IV criteria for IBS. Participants are randomized (1:1), stratified by IBS subtype and gender, to either the WISH 2.0 intervention or an educational control condition. Both conditions consist of 9 weekly 30-minute phone sessions accompanied by weekly manualized exercises. Primary outcomes are feasibility (proportion completing ≥6 of 9 sessions) and acceptability (weekly ease and utility ratings). Secondary outcomes include WISH 2.0’s preliminary effects on health-related, health behavior, and psychological outcomes, assessed at 4 time points: baseline, intervention midpoint, the postintervention assessment, and the 3-month postintervention assessment. Candidate gut-brain mechanisms that might underlie WISH 2.0’s effects, including changes in autonomic function (heart rate variability), interoception (self-report measure), gene expression (whole transcriptome RNA sequencing), and immune system activity (serum inflammatory biomarkers), will be explored before and after the intervention.

**Results:**

The trial was approved by the Mass General Brigham Institutional Review Board and prospectively registered at ClinicalTrials.gov in March 2025. Funding was awarded in June 2025. Following a study start-up period, recruitment commenced in October 2025 and is ongoing. As of July 2026, 20 participants have been enrolled. Study completion is anticipated in August 2028.

**Conclusions:**

This trial will determine the feasibility and acceptability of WISH 2.0, a novel PP intervention for IBS. Comparative results between WISH 2.0 and an educational control, including the possibility of equivalent outcomes, will provide important mechanistic insights and facilitate further optimization of BGBTs for IBS, informing a future fully powered efficacy trial.

## Introduction

Irritable bowel syndrome (IBS) is one of the most common disorders of gut-brain interaction (previously known as functional gastrointestinal [GI] disorders) [1], affecting approximately 10% to 15% of adults worldwide [2,3]. It is associated with significant psychiatric comorbidity [4], health care costs [5], and impairments in health-related quality of life [6]. It has no known structural causes or biomarkers that can be used for diagnosis and is instead understood to arise from complex interactions among biological, psychological, and social factors [3,7].

Behavioral interventions designed to mitigate GI symptoms, known as brain-gut behavior therapies (BGBTs; eg, GI-focused cognitive behavioral therapy), represent an important treatment option for IBS [3,8]. Like many behavioral interventions, however, existing BGBTs for IBS face limitations related to scalability (due to the need for specialized behavioral health providers) [9-11] and acceptability and patient preference (due to perceived stigma of a psychological intervention and/or lack of relevance to IBS symptoms) [11,12]. Additionally, existing BGBTs do not specifically focus on boosting *positive psychological (PP) well-being*, which has been linked to a variety of health outcomes [13].

Increasingly recognized as an important contributor to both mental and physical health in IBS [14,15], PP well-being encompasses multiple adaptive PP constructs, including resilience, optimism, and self-efficacy [16]. More than just the absence of psychopathology, PP well-being represents a distinct dimension of whole-person health [17,18]. Compared with healthy populations, individuals with IBS consistently report lower overall well-being [19-21], as well as lower levels of specific PP constructs, including resilience [20,22-24], positive affect [25], and self-efficacy [24,26]. Lower well-being has in turn been linked to greater IBS symptom severity [22,23,27], heightened depressive [27-30] and anxiety symptoms [27,29,30], and reduced health-related quality of life [22,27,30,31]. Conversely, there is evidence in the general population [13] and other medically ill populations (eg, cardiovascular disease [32,33]) that greater well-being may be prospectively linked to more favorable clinical outcomes, such as slower disease progression and reduced all-cause mortality risk [13,32,34].

Aside from our prior proof-of-concept work [35], interventions to specifically boost PP well-being in IBS have not yet been investigated and represent a much-needed area of future research [15]. PP interventions consist of brief, structured exercises designed to enhance well-being. Across both healthy and chronically ill populations, these interventions have demonstrated promising improvements in well-being [17,18], reductions in perceived stress and depressive symptoms [17,18], greater adherence to health-related behaviors [36-44], and improvements in specific clinical outcomes, such as pain [45]. Furthermore, PP interventions have the potential to overcome some of the limitations associated with existing BGBTs for IBS: they are highly scalable (they can be delivered remotely [44] and administered by bachelor’s-level interventionists [44,46]), and they tend to be well received and perceived as nonstigmatizing [44,46], which may enhance engagement and uptake.

Therefore, informed by the Rome Foundation Consensus Statement on the design of BGBT clinical trials [47], the ORBIT (Obesity-Related Behavioral Intervention Trials) model of behavioral treatment development for chronic diseases [48], and the National Institutes of Health (NIH) stage model for behavioral intervention development [49], and building on prior qualitative research [50] and a systematic review [14], we developed the WISH (Well-being in IBS: Strengths and Happiness) program, a novel, 9-week, manualized, phone-delivered PP intervention specifically adapted to IBS. We examined its feasibility, acceptability, and potential effects on key clinical outcomes in a prior proof-of-concept randomized waitlist-controlled trial (N=22) [35]. WISH was found to be preliminarily feasible (85% of all sessions were completed, exceeding our a priori threshold of 67%), easy to complete (mean 7.2 out of 10, 95% CI 6.70-7.75), and subjectively helpful (mean 7.6 out of 10, 95% CI 7.14-8.01). Promising signals for improvement on several clinically meaningful outcomes, including IBS symptom severity, IBS health-related quality of life, resilience, GI symptom-specific anxiety, restrictive and avoidant eating, perceived stress, and pain catastrophizing, were identified, with effect sizes (Cohen *d*) ranging from 0.1 to 0.7. Of particular note, 61% (11/18) of participants no longer met Rome IV criteria [51] for IBS after intervention completion. Though this proof-of-concept trial provided promising initial data regarding WISH, it had a small sample, no active control condition, no extended follow-up to assess sustainability of effects, and no examination of potential underlying gut-brain mechanisms, the latter of which is often missing from BGBT clinical trials. Additionally, participant feedback highlighted areas for further refinement, including greater customization of intervention content to IBS and expanded descriptions of connections between PP exercises and the gut-brain axis.

Accordingly, we now seek to evaluate the feasibility, acceptability, preliminary effects, and candidate gut-brain mechanisms of an optimized and refined version of WISH (WISH 2.0) in a randomized controlled trial (RCT; N=50) compared with a time- and attention-matched educational control.

## Methods

### Objectives

The primary aim of this RCT is to evaluate the feasibility, acceptability, and preliminary effects of an optimized 9-week, phone-delivered, manualized PP intervention for IBS (WISH 2.0), compared with a time- and attention-matched educational control condition. The secondary aim is to explore candidate gut-brain mechanisms through which WISH 2.0 might impact IBS symptoms.

### Hypotheses

#### Aim 1: Feasibility, Acceptability, and Preliminary Effects

We hypothesize that WISH 2.0 will be feasible and acceptable. Feasibility will be defined as at least 50% of participants completing ≥6 of 9 intervention sessions. Acceptability will be defined as mean ratings of ≥7 on a 10-point Likert scale assessing exercise ease and utility (0=not at all; 10=very much). We further hypothesize that, compared with the educational control condition, a greater proportion of participants randomized to WISH 2.0 will achieve a clinically meaningful improvement in symptoms, defined as ≥50-point reduction from baseline on the IBS Symptom Severity Scale (IBS-SSS) [52] (ie, the minimal clinically important difference [MCID]). In addition, WISH 2.0 will be associated with small-to-medium effect size improvements in IBS symptom severity, as well as in other exploratory psychological and health-related outcomes.

#### Aim 2: Candidate Gut-Brain Mechanisms

We hypothesize that participation in WISH 2.0 will be associated with changes in at least one of the following candidate gut-brain mechanisms: autonomic function (heart rate variability [HRV]), interoception (self-report measure), stress-related gene expression (whole transcriptome RNA sequencing), and immune system activation (serum inflammatory biomarkers). We further hypothesize that any changes in these candidate mechanisms will be associated with reductions in IBS symptom severity over time.

### Study Design

This study is a single-site, parallel-group pilot RCT that aims to enroll 50 adults with IBS. Participants are randomized 1:1 to WISH 2.0 or a time- and attention-matched educational control condition. The intervention period is 9 weeks, with extended follow-up at 3 months after the intervention. The trial was prospectively registered at ClinicalTrials.gov (NCT06866106). This protocol is reported in accordance with the SPIRIT (Standard Protocol Items: Recommendations for Interventional Trials) 2025 statement (Checklist 1), the CONSORT (Consolidated Standards of Reporting Trials) 2025 statement [53], and the CONSORT extension for pilot and feasibility trials [54].

### Ethical Considerations

The study was approved by the Mass General Brigham (MGB) Institutional Review Board (IRB; 2025P000594) in March 2025 prior to the initiation of any study activities. Any protocol amendments have, and will be, submitted to the IRB for approval prior to implementation and communicated to trial registries and relevant parties as appropriate. Participants provide informed consent prior to enrollment; consent procedures are further described in the Assessment Visits section. The privacy and confidentiality of the research participants’ data and identity are maintained. Participants receive US $50 for completing each of the 4 assessment time points (maximum total compensation: US $200) and are reimbursed for parking at the 2 in-person visits (see Assessment Visits section). An independent data and safety monitoring board (DSMB), comprising 4 senior investigators recognized for their expertise in clinical trials methodology, biostatistics, clinical gastroenterology, and gastropsychology and behavioral medicine, respectively, was appointed to review and evaluate study data for participant safety, study conduct, and study progress. Adverse events and serious adverse events are systematically monitored throughout the trial and reported to the IRB and DSMB. The DSMB reviews cumulative safety data at regular intervals and provides expedited review for any serious adverse events, and has the authority to recommend suspension or termination of the trial based on safety. The DSMB convened for its first meeting prior to study initiation.

### Study Development

#### PP Intervention

**Figure 1.**
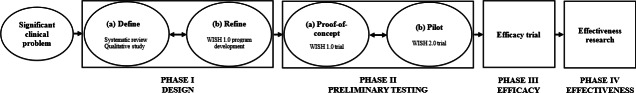
Framework for intervention development. WISH: Well-being in IBS: Strengths and Happiness.

Development of the WISH program was informed by the Rome Foundation Consensus Statement on the design of BGBT clinical trials [47], the ORBIT model of behavioral treatment development for chronic diseases [48], and the NIH stage model for behavioral intervention development [49] (Figure 1). Each of these models of behavioral intervention development emphasizes a flexible, iterative approach inclusive of early pre-efficacy study phases. The WISH intervention is based on PP interventions designed for other chronic medical conditions, including heart disease, obesity, type 2 diabetes, and cancer, among others [38,41-44]. In preparation to specifically adapt the PP intervention for the unique experiences of individuals with IBS, both a systematic review [14] and a qualitative study [50] were first completed (ORBIT phase Ia: define). Results from these studies informed the adaptation and development of the original WISH intervention (ORBIT phase Ib: refine), which was evaluated in a proof-of-concept, randomized waitlist-controlled trial (N=22) [35] with qualitative exit interviews to facilitate subsequent refinement of study procedures and intervention content (ORBIT phase IIa: proof-of-concept). These refinements, including greater customization of the content to IBS, expanded descriptions of the connections between PP exercises and the gut-brain axis, and replacement of the lowest-rated exercise, resulted in WISH 2.0, which is now being tested in the described RCT (ORBIT phase IIb: pilot testing) [48]. Of note, the most recent iteration of the ORBIT model would likely classify this trial as a hybrid of phase Ib (refine), given its exploration of candidate gut-brain mechanisms, and phase IIa (proof-of-concept), as it aims to evaluate whether WISH 2.0 can produce a plausible clinically significant benefit [55].

#### Educational Control

Education is a common comparator condition used to control for therapist attention, contact, and support, as well as participant expectancy in behavioral intervention trials [56]. This phone-based, manualized educational control program was designed to match the WISH 2.0 intervention in terms of therapist contact, attention, and time. Participants’ perceptions of programmatic credibility and expectations for treatment are measured using the Credibility/Expectancy Questionnaire (CEQ) [57] following the first phone session of both programs. The manual and accompanying phone sessions provide information about the history, pathophysiology, and treatment of IBS, but importantly do not include active therapeutic components, provide active skills training, or promote the enhancement of PP constructs. This 9-week educational control program was adapted, with permission, from two 8-week educational control programs for individuals with functional dyspepsia and fibromyalgia [58], respectively. The approach to adaptation was also partially informed by a 4-week educational control program for IBS [59].

### Participants

We plan to enroll and randomize 50 participants. Inclusion criteria are age ≥18 years, IBS diagnosis meeting Rome IV criteria (as assessed by the Rome IV Diagnostic Questionnaire—IBS Module [51] and confirmed in the electronic medical record), English fluency, and access to a telephone. Exclusion criteria are presence of inflammatory bowel disease or other primary, structural gastrointestinal disease that better explains IBS-like symptoms; severe psychiatric illness (current mania, psychosis, or active substance use disorder, assessed via the Mini International Neuropsychiatric Interview [60] or the Diagnostic Interview for Anxiety, Mood, and Obsessive-Compulsive Disorder and Related Neuropsychiatric Disorders [61]) or cognitive impairment (assessed via a 6-item screen [62]) that might preclude participation; atrial fibrillation, pacemaker, or daily β-blocker use, due to effects on HRV testing; and confounding plans to initiate psychotherapy or another behavioral health intervention during the concurrent study period.

### Recruitment

Participants are recruited primarily from the MGB clinical referral network in the greater Boston area via three main strategies: (1) the Research Patient Data Registry, a centralized clinical data registry that gathers and consolidates clinical information from across the MGB hospital network [63]; (2) MGB gastroenterology or primary care clinician referral (either directly, via a fact sheet distributed to relevant clinics, or via the High Enroll clinical trial recruitment platform for clinicians); and (3) the MGB Rally online research recruitment platform designed to connect community members with ongoing clinical research studies. Potential participants identified from the Research Patient Data Registry, clinician referral, and prior research studies are sent research invitation letters and study fact sheets by mail or through their patient portals. The research invitation briefly describes the study, the procedure to opt out of future contact, and whom to call for further information. The study fact sheet summarizes the study information in greater detail. The letter and fact sheet are followed by a recruitment screening phone call 7 days later for those who do not opt out of further contact. Potential participants who express interest in the study after learning about it from the MGB Rally online platform are contacted by phone to complete screening phone calls, as described below. Recruitment will cease when 50 participants have been randomized, or if the DSMB recommends stopping due to safety concerns. Screening and recruitment data, including numbers of individuals identified, contacted, screened, eligible, enrolled, and reasons for ineligibility or nonparticipation, will be tracked prospectively and reported in accordance with the CONSORT 2025 statement [53] and CONSORT extension for pilot and feasibility trials [54].

### Participant Screening

All potential participants complete a screening phone call for the previously described inclusion and exclusion criteria, including IBS diagnosis meeting Rome IV criteria (per the Rome IV Diagnostic Questionnaire—IBS Module [51]). Prior to the screening phone call, potential participants have already received the study fact sheet or reviewed the study information on the MGB Rally online research recruitment platform. In addition to the screening phone call, the electronic health record is reviewed to confirm IBS diagnosis and other inclusion and exclusion criteria. If insufficient documentation is identified in the electronic medical record to confirm IBS diagnosis, potential participants are asked to provide a release of information to obtain further records from their medical providers. All recruitment emphasizes voluntariness and nonimpact on clinical care. Once eligibility is confirmed, participants are scheduled for an in-person baseline visit that includes obtaining informed signed consent.

### Assessment Visits

Participants complete 4 assessments: baseline (in-person), intervention midpoint (remote), the postintervention assessment (in-person), and the 3-month postintervention assessment (remote). Written informed consent is obtained electronically via a secure REDCap platform at the baseline visit. At each assessment, participants complete a set of self-report questionnaires electronically via a secure REDCap link. In-person assessments are conducted at the hospital’s Translational and Clinical Research Center. At the 2 in-person visits (ie, at baseline and immediately after the intervention), participants also provide a blood sample for whole transcriptome and serum inflammatory biomarker analyses and undergo HRV assessment with the emWave Pro Plus (HeartMath Institute), a validated photoplethysmography ear-clip sensor [64,65]. HRV is measured for a total of 9 minutes while participants complete a set of standardized tasks (5 min of resting at baseline, 3 min of focused attention, and 1 min of deep breathing). Additionally, participants complete a brief exit interview as part of the postintervention assessment.

### Randomization and Blinding

At the conclusion of the baseline visit, after providing informed consent and completing all baseline assessments (ie, questionnaires, HRV measurement, and blood sample collection), participants are randomized, stratified by IBS subtype and gender, using the REDCap randomization module to either the PP or educational control program. The randomization sequence was generated by the senior author (CMC) using permuted blocks of variable size, with stratification by IBS subtype (IBS with predominant constipation, IBS with predominant diarrhea, IBS with mixed bowel habits, or unclassified IBS) and gender (male, female, or nonbinary). Allocation concealment is ensured with the REDCap randomization module, which withholds the allocation sequence until after the participant has provided informed consent and completed baseline assessments. After randomization, participants are provided with the appropriate manual. Participants are masked to study hypotheses (ie, whether their randomly assigned program is the intervention or the control), and outcome assessors are masked to treatment assignment.

### Study Interventions

#### WISH 2.0 Intervention

WISH 2.0 is a 9-week, manualized PP intervention delivered via weekly phone sessions lasting approximately 30 minutes with a trained study interventionist (primarily a psychiatrist [ENM]). Each week, participants are assigned structured readings and worksheets from the intervention manual designed to support practice of a specific PP skill. During the weekly sessions, the interventionist reviews the prior week’s activity with the participant and introduces the subsequent week’s exercise. The content and sequence of weekly topics are summarized in Table 1.

**Table 1.** WISH (Well-being in IBS: Strengths and Happiness) positive psychological (PP) intervention.

Week	Intervention	Description
1	Gratitude for positive events [66]	Recall 3 positive events from the preceding week
2	Using personal strengths [67]	Select a characteristic personal strength and use it in a new way
3	Promoting variety and meaning [68]	Complete 3 variations of enjoyable and meaningful activities
4	Capitalizing on positive events [67]	Identify 3 positive events and capitalize on them in specific ways
5	Positive reappraisal [69]	Reframe negative events and identify adaptive qualities gained
6	Recalling a past success [70]	Recall a past success and the skills it required
7	Perseverance [67]	Use the skill of perseverance to achieve a goal
8	The good life [71]	Use the skill of optimism to envision a future “good life” in 1 year
9	Planning for the future	Create a plan to continue using newly learned PP skills after the program

#### Educational Control Condition

The time- and attention-matched educational control condition is a 9-week, manualized program delivered via weekly phone sessions lasting approximately 30 minutes with the same trained study interventionist as the WISH 2.0 intervention. Each week, participants are assigned structured educational readings and accompanying worksheets designed to reinforce comprehension of the material. During the weekly sessions, the interventionist reviews the prior week’s content and completed worksheets with the participant and introduces the subsequent week’s assignment. The content and sequence of weekly topics are summarized in Table 2.

**Table 2.** Educational control condition.

Week	Topic
1	IBS^a^ basics
2	History of IBS
3	Biopsychosocial model overview
4	Biological factors 1: gut permeability, motility, visceral hypersensitivity, microbiome, and inflammation
5	Biological factors 2: genetics, autonomics, interoception, intolerances, infection, and neurotransmitters
6	Psychological and social factors: psychiatric and psychological comorbidity, support, and stress
7	Treatment options: education, lifestyle, medication, psychological and behavioral, and complementary therapies
8	Patient-clinician relationship
9	Program reflections

a IBS: irritable bowel syndrome.

### Intervention Delivery and Fidelity

The primary study interventionist is a psychiatrist (ENM). Phone sessions are audio-recorded, and a subset are reviewed for fidelity by a study team psychologist using fidelity scales specifically developed for each treatment arm to ensure consistency within and across interventions. Cases are reviewed and discussed regularly.

### Outcomes

The timing of primary and secondary outcome assessments is summarized in Table 3.

**Table 3.** Schedule of assessments of primary and secondary outcomes.

	Baseline	Week 1	Week 2	Week 3	Week 4	Week 5	Week 6	Week 7	Week 8	Week 9	Extended follow-up
Primary outcomes											
Feasibility											
Percentage of PP sessions completed										✓	
Acceptability											
Ease and utility ratings			✓	✓	✓	✓	✓	✓	✓	✓	
Exit interview										✓	
Secondary outcomes											
Credibility and expectancy											
Self-report measure		✓									
Immediate impact of exercises											
Likert scales			✓	✓	✓	✓	✓	✓	✓	✓	
Preliminary effects											
Self-report measures	✓					✓				✓	
Exploratory mechanisms											
Heart rate variability	✓									✓	
Self-reported interoception (MAIA-2^a^)	✓					✓				✓	✓
Blood whole transcriptome analysis	✓									✓	
Serum inflammatory biomarker analysis	✓									✓	

a MAIA-2: Multidimensional Assessment of Interoceptive Awareness Version 2.

#### Primary Outcomes

The primary outcomes of this study are feasibility and acceptability. Feasibility of the intervention is assessed by the proportion of sessions completed. A session is recorded as completed if the participant performs and then reviews the PP exercise with the interventionist during a phone session. The intervention will be considered feasible if at least 50% of participants complete ≥6 of 9 PP sessions, consistent with prior PP-based intervention feasibility trials [40-44,72]. If a participant drops out, all subsequent exercises will be considered incomplete. Acceptability is assessed using participant ratings of ease (“How easy was it to complete the exercise?”) and utility (“Overall, how helpful do you feel the exercise was?”) of each intervention topic on a scale from 0 to 10 (0=not at all, 10=extremely). The intervention will be considered well accepted if mean ease and utility ratings are ≥7 of 10, based on similar metrics in prior PP studies [40-44,72]. Semistructured qualitative exit interviews assessing acceptability (eg, most and least helpful sessions, ease, program structure, and delivery) will also be completed.

#### Credibility and Expectancy of Each Program

Participants in both treatment arms will complete the CEQ [57] after their first phone session to assess perceived program credibility and expectations for benefit.

#### Immediate Impact of Weekly Activities

To examine the immediate impact of the respective programs, participants in the PP intervention will be asked to rate levels of happiness and optimism before and after each PP exercise on 10-point Likert scales (0=not happy or optimistic; 10=very happy or optimistic). Participants in the educational control condition will be asked to rate their level of knowledge about IBS before and after each educational exercise on a 10-point Likert scale (0=not knowledgeable; 10=very knowledgeable). Changes from before to after the exercises on these scales will be considered to represent the immediate impact of the respective exercises on these constructs.

#### Exploratory Clinical Outcomes

The comparative effects of the interventions on several health-related, psychological, and health behavior outcomes will be explored at baseline, intervention midpoint, immediately after the intervention, and 3 months after the intervention (ie, extended follow-up). Health-related outcomes include IBS symptom severity (IBS-SSS; primary exploratory clinical outcome) [52], health-related quality of life (IBS Quality of Life Instrument [IBS-QOL] [73]), and IBS diagnosis (Rome IV Diagnostic Questionnaire—IBS Module) [51]. Consistent with the literature [52,74], the MCID for within-participant change from baseline for this trial will be defined a priori as a reduction of ≥50 points in the IBS-SSS total score at the postintervention assessment. Psychological outcomes include positive affect (Positive and Negative Affect Schedule [PANAS] [75]), optimism (Life Orientation Test-Revised [LOT-R] [76] and the Brief State Optimism Measure [B-SOM] [77]), resilience (Brief Resilience Scale [BRS] [78]), self-efficacy (General Self-Efficacy Scale [GSE] [79]), depression and anxiety (Hospital Anxiety and Depression Scale [HADS] [80]), GI symptom-specific anxiety (Visceral Sensitivity Index [VSI] [81]), response to pain (the Pain Catastrophizing Scale [PCS] [66]), and perceived stress (the Perceived Stress Scale (PSS) [82]). Health behavior outcomes include physical activity (International Physical Activity Questionnaire [IPAQ] [83]) and avoidant and restrictive eating (Nine Item Avoidant/Restrictive Food Intake Disorder Screen (NIAS) [84]).

#### Exploratory Mechanistic Outcomes

Exploratory mechanistic outcomes include autonomic function (as measured by HRV frequency-domain variable measures, specifically low-frequency power, high-frequency power, and the low-frequency/high-frequency ratio [85,86], using the HeartMath Institute emWave Pro Plus), self-reported interoception (Multidimensional Assessment of Interoceptive Awareness Version 2 [MAIA-2] [87]), stress-mediated gene expression (whole transcriptome analysis of blood by RNA sequencing), and immune system activation (serum inflammatory biomarker levels, such as interleukin-6, tumor necrosis factor-α, and C-reactive protein). Each of these 4 candidate gut-brain mechanisms is known to contribute to IBS pathophysiology (at least in subsets of patients) [88-99] and has established associations with PP well-being [100-104]; thus, each holds promise as a potential mechanism linking PP well-being and IBS symptom severity. These exploratory mechanisms will largely be assessed before and immediately after the intervention, except for interoception, as this is measured by a questionnaire that can be completed remotely at the intervention midpoint and extended follow-up.

### Analytical Approach

#### Power and Sample Size

This pilot trial (N=50) is powered to evaluate feasibility and acceptability and is not designed to detect statistically significant between-group differences in exploratory clinical or mechanistic outcomes. Based on our prior proof-of-concept trial [35], with 25 participants in the PP intervention, we will have 90% power to detect a difference between our expected proportion of 80% of participants completing at least 6 (67%) of 9 PP activities and the null hypothesis (true proportion of 50% or less) at the 2-sided .05 significance level using a proportion test (power oneproportion routine in Stata version 17; StataCorp LLC). Similarly, for acceptability, assuming the same mean ratings as in our prior proof-of-concept trial [35] and an average of 6 measurements from the 25 PP intervention participants, this study has more than 90% power to detect a mean acceptability rating of ≥7.0 of 10 (simulation-based power calculation). Regarding the exploratory mediation analyses, with our sample size of 50 participants, we will have 81% power to detect a mediation effect of at least 0.44 assuming a standardized mediator with SD of 1, a standardized outcome, and a correlation between the treatment and the mediator of 0.4 at the 2-sided .05 significance level (powerMediation.VSMc command from the *powerMediation* library in R; R Foundation for Statistical Computing). Although we do not anticipate finding significant mediation effects in this pilot trial, these analyses may help identify promising candidate gut-brain mechanisms that merit examination in a future, larger randomized efficacy trial.

#### Statistical Analysis Plan

Feasibility will be assessed using proportions and exact binomial CIs. Acceptability will be evaluated using a random-effects model with a participant-specific random effect to account for within-participant correlation among ratings. This model will also facilitate estimation of the between- and within-participant variance of the ratings. Qualitative exit interviews will be coded by 2 independent coders with a goal of interrater reliability >0.80. Discrepancies will be resolved by consensus, and themes will be derived from iterative review of coded data. To assess the immediate impact of the respective exercises on happiness, optimism, and IBS knowledge, 2-tailed paired *t* tests will be used. Between-group differences in the proportion who achieve the MCID (≥50-point reduction in the IBS-SSS) will be evaluated using logistic regression. Achieving the MCID (yes or no) will be modeled as the dependent variable, with treatment group (WISH 2.0 vs educational control) included as the primary independent variable. For clinical outcomes that are measured multiple times per participant, preliminary effects will be explored using mixed-effects regression models with fixed effects for a categorical effect of time and a time-by-treatment interaction with a common baseline for the 2 groups, and an unstructured covariance matrix for the residuals to account for repeated measures. The time-by-treatment interaction terms will estimate the difference between groups in the change over time. Because the mixed-effects model uses maximum likelihood to estimate the coefficients, this approach is valid when missing data are missing at random. Effect sizes (modified Cohen *d*) will be calculated by dividing the group×time coefficient by the SD of the difference estimated using the appropriate variance components from the unstructured covariance matrix. Exploratory mechanistic analyses will use linear mixed models to assess changes in candidate gut-brain mechanisms (eg, HRV, interoception, gene expression, and immune activity) in response to the intervention. If there are skewed distributions in the candidate mechanistic data, logarithmic transformations will be applied. Given the exploratory nature of these analyses, no correction for multiple comparisons will be used. In addition to the models that examine each outcome separately, we will also preliminarily explore whether these gut-brain mechanisms might mediate IBS symptom change using a mediation model. We will calculate the change in the candidate mechanism measurements using linear regression for each participant and the change in the clinical effects outcome measures. Then, we will fit a mediation model with the change in the clinical effects outcome measures as the outcome, the change in the mechanistic measurements as the mediator, and the treatment group as the predictor. The direct and indirect effects of the treatment on the clinical effects outcome measures will be estimated from this approach. Finally, the mediation effects will also be estimated by calculating the change in the treatment effect from the linear mixed model used to assess preliminary clinical effects and the treatment effect from a linear mixed model including treatment, time, time-by-treatment interaction, baseline mechanistic data, and change in mechanistic data as the predictors. The change will show the indirect effect through changes in candidate gut-brain mechanisms. All analyses will be conducted using Stata (version 17). Analyses of clinical and candidate mechanistic outcomes will be conducted on the intention-to-treat population (all randomized participants), with sensitivity analyses using the per-protocol population where appropriate.

## Results

Prior to participant enrollment, IRB approval was obtained and the trial was prospectively registered at ClinicalTrials.gov (NCT06866106) in March 2025. Funding was awarded in June 2025, and following a study start-up period, recruitment commenced in October 2025. As of July 2026, 20 participants have enrolled in the study. Study completion is anticipated in August 2028; at that time, findings related to feasibility, acceptability, and preliminary clinical and mechanistic outcomes will be reported.

## Discussion

### Anticipated Findings

The WISH 2.0 RCT will evaluate the feasibility, acceptability, and preliminary effects of a novel, iteratively refined, phone-delivered PP intervention for IBS and will explore candidate gut-brain mechanisms that might underlie its potential effects. This intervention extends existing BGBTs by explicitly targeting PP well-being, an underexplored yet important contributor to IBS symptom burden, mental health, and health-related quality of life [14,50].

There are several strengths to the WISH 2.0 clinical trial. These include a systematic, evidence-based approach to behavioral intervention and BGBT development; a randomized design with an active and clinically meaningful time- and attention-matched control condition; masking of participants to hypotheses and assessors to treatment condition; processes to ensure intervention fidelity; a priori specified outcomes and use of validated measures; and the exploratory integration of multimodal mechanistic assessments spanning autonomic function, self-reported interoception, gene expression, and immune activation. Exploration of candidate mechanisms early in behavioral intervention development is key to facilitating more focused mechanistic examinations in future larger trials. In addition, the remote delivery format and relative ease of administration (eg, potential delivery by bachelor’s-level interventionists [44,46]) enhance scalability and accessibility, which may facilitate future implementation in real-world clinical settings.

There are also limitations to this study. As a single-site pilot trial with a relatively small sample size, it is not powered to detect statistically significant differences in clinical outcomes or candidate mechanisms; accordingly, analyses of effects and mechanisms will be exploratory. Additionally, although the educational control condition is designed to match time, attention, and credibility, nonspecific factors may still influence outcomes.

If WISH 2.0 is found to be feasible and acceptable and demonstrates promising signals of benefit, these findings will inform the design of a fully powered RCT. In addition, exploratory mechanistic findings may help identify candidate pathways through which PP interventions might influence IBS symptoms, supporting further refinement, optimization, and mechanistic targeting of BGBTs for the IBS population.

### Conclusions

This RCT will provide critical data regarding the feasibility and acceptability of WISH 2.0, a novel PP intervention for IBS. Comparative findings between WISH 2.0 and an educational control condition—including the possibility of equivalent effects—will offer important insights into BGBT mechanisms. These results have the potential to support the refinement and development of scalable, mechanistically targeted BGBTs for IBS and to inform the design of future large-scale trials.

## Supplementary material

10.2196/98352 Checklist 1 SPIRIT 2025 checklist.

## Abbreviations

B-SOM: Brief State Optimism Measure
BGBT: brain-gut behavior therapy
BRS: Brief Resilience Scale
CEQ: Credibility/Expectancy Questionnaire
CONSORT: Consolidated Standards of Reporting Trials
DSMB: data and safety monitoring board
GI: gastrointestinal
GSE: General Self-Efficacy Scale
HADS: Hospital Anxiety and Depression Scale
HRV: heart rate variability
IBS: irritable bowel syndrome
IBS-QOL: Irritable Bowel Syndrome Quality of Life Instrument
IBS-SSS: Irritable Bowel Syndrome Symptom Severity Scale
IPAQ: International Physical Activity Questionnaire
IRB: institutional review board
LOT-R: Life Orientation Test-Revised
MAIA-2: Multidimensional Assessment of Interoceptive Awareness Version 2
MCID: minimal clinically important difference
MGB: Mass General Brigham
NIAS: Nine Item Avoidant/Restrictive Food Intake Disorder Screen
NIH: National Institutes of Health
ORBIT: Obesity-Related Behavioral Intervention Trials
PANAS: Positive and Negative Affect Schedule
PCS: Pain Catastrophizing Scale
PP: positive psychological
RCT: randomized controlled trial
SPIRIT: Standard Protocol Items: Recommendations for Interventional Trials
VSI: Visceral Sensitivity Index
WISH: Well-Being in IBS: Strengths and Happiness
