# Supplementary material for: The WISH 2.0 Intervention for Irritable Bowel Syndrome: Protocol for a Pilot Randomized Controlled Trial
Source: JMIR Res Protoc. 2026 Jul 23;15:e98352. doi: 10.2196/98352 (PMC13395429; doi:10.2196/98352)
Supplement: Checklist 1 [file resprot-v15-e98352-s001.pdf]

Table 1. SPIRIT 2025 Checklist

Section/Topic	Item No.	SPIRIT 2025 Checklist Item Description	Reported in Section
**Administrative information**			
Title and structured summary	**1a**	Title stating the trial design, population, and interventions, with identification as a protocol	Title page
	**1b**	Structured summary of trial design and methods, including items from the WHO Trial Registration Data Set	Abstract
Protocol version	**2**	Version date and identifier	Ethics Approval
Roles and responsibilities	**3a**	Names, affiliations, and roles of protocol contributors	Title page
	**3b**	Name and contact information for the trial sponsor	Funding
	**3c**	Role of trial sponsor and funders in design, conduct, analysis, and reporting of trial; including any authority over these activities	Funding
	**3d**	Composition, roles, and responsibilities of the coordinating site, steering committee, endpoint adjudication committee, data management team, and other individuals or groups overseeing the trial, if applicable	Ethics Approval
**Open science**			
Trial registration	**4**	Name of trial registry, identifying number (with URL), and date of registration	Abstract; Results
Protocol and statistical analysis plan	**5**	Where the trial protocol and statistical analysis plan can be accessed	Results
Data sharing	**6**	Where and how the individual de-identified participant data (including data dictionary), statistical code, and any other materials will be accessible	Data Availability
Funding and conflicts of interest	**7a**	Sources of funding and other support (e.g., supply of drugs)	Funding
	**7b**	Financial and other conflicts of interest for principal investigators and steering committee members	Disclosures
Dissemination policy	**8**	Plans to communicate trial results to participants, healthcare professionals, the public, and other relevant groups	Discussion; Results
**Introduction**			
Background and rationale	**9a**	Scientific background and rationale, including summary of relevant studies (published and unpublished) examining benefits and harms for each intervention	Introduction
	**9b**	Explanation for choice of comparator	Methods - Study Development/Educational Control
Objectives	**10**	Specific objectives related to benefits and harms	Methods - Objectives; Hypotheses
**Methods: Patient and public involvement, trial design**			
Patient and public involvement	**11**	Details of, or plans for, patient or public involvement in the design, conduct, and reporting of the trial	Methods - Study Development
Trial design	**12**	Description of trial design including type of trial (e.g., parallel group, crossover), allocation ratio, and framework (e.g., superiority, equivalence, non-inferiority, exploratory)	Methods - Study Design
**Methods: Participants, interventions, and outcomes**			

Trial setting	**13**	Settings and locations where the trial will be conducted	Methods - Participants; Assessment Visits
Eligibility criteria	**14a**	Eligibility criteria for participants	Methods - Participants
	**14b**	If applicable, eligibility criteria for sites and for individuals who will deliver the interventions (e.g., surgeons, physiotherapists)	Methods - Intervention Delivery and Fidelity
Intervention and comparator	**15a**	Intervention and comparator with sufficient details to allow replication including how, when, and by whom they will be administered	Methods - Study Interventions; Tables 2-3
	**15b**	Criteria for discontinuing or modifying allocated intervention/comparator for a trial participant	Ethics Approval and Data and Safety Monitoring
	**15c**	Strategies to improve adherence to intervention/comparator protocols, and any procedures for monitoring adherence	Methods - Primary Outcomes; Intervention Delivery and Fidelity
	**15d**	Concomitant care that is permitted or prohibited during the trial	Methods - Participants (exclusion criteria)
Outcomes	**16**	Primary and secondary outcomes, including the specific measurement variable, analysis metric, method of aggregation, and time point for each outcome	Methods - Outcomes; Table 4
Harms	**17**	How harms are defined and will be assessed (e.g., systematically, non-systematically)	Methods - Ethics Approval and Data and Safety Monitoring; Intervention Delivery and Fidelity
Participant timeline	**18**	Time schedule of enrollment, interventions (including any run-ins and washouts), assessments, and visits for participants. A schematic diagram is highly recommended	Methods - Table 4 (Schedule of Assessments)
Sample size	**19**	How sample size was determined, including all assumptions supporting the sample size calculation	Methods - Power and Sample Size
Recruitment	**20**	Strategies for achieving adequate participant enrollment to reach target sample size	Methods - Recruitment
**Methods: Assignment of interventions**			
Randomization: Sequence generation	**21a**	Who will generate the random allocation sequence and the method used	Methods - Randomization and Blinding
	**21b**	Type of randomization (simple or restricted) and details of any factors for stratification	Methods - Randomization and Blinding
Allocation concealment mechanism	**22**	Mechanism used to implement the random allocation sequence (e.g., central computer/telephone; sequentially numbered, opaque, sealed containers), describing any steps to conceal the sequence until interventions are assigned	Methods - Randomization and Blinding
Implementation	**23**	Whether the personnel who will enroll and those who will assign participants to the interventions will have access to the random allocation sequence	Methods - Randomization and Blinding
Blinding	**24a**	Who will be blinded after assignment to interventions (e.g., participants, care providers, outcome assessors, data analysts)	Methods - Study Design; Randomization and Blinding
	**24b**	If blinded, how blinding will be achieved and description of the similarity of interventions	Methods - Study Design; Randomization and Blinding
	**24c**	If blinded, circumstances under which unblinding is permissible, and procedure for revealing a participant's allocated intervention during the trial	Not applicable

**Methods: Data collection, management, and analysis**			
Data collection methods	**25a**	Plans for assessment and collection of trial data, including any related processes to promote data quality and a description of trial instruments along with their reliability and validity, if known	Methods - Outcomes; Assessment Visits
	**25b**	Plans to promote participant retention and complete follow-up, including list of any outcome data to be collected for participants who discontinue or deviate from intervention protocols	Methods - Intervention Delivery and Fidelity; Primary Outcomes
Data management	**26**	Plans for data entry, coding, security, and storage, including any related processes to promote data quality	Methods - Assessment Visits (REDCap)
Statistical methods	**27a**	Statistical methods used to compare groups for primary and secondary outcomes, including harms	Methods - Analytical Approach
	**27b**	Definition of who will be included in each analysis and in which group	Methods - Analytical Approach
	**27c**	How missing data will be handled in the analysis	Methods - Analytical Approach
	**27d**	Methods for any additional analyses (e.g., subgroup and sensitivity analyses)	Methods - Analytical Approach
**Methods: Monitoring**			
Data monitoring committee	**28a**	Composition of data monitoring committee (DMC); summary of its role and reporting structure; statement of whether it is independent from the sponsor and funder; conflicts of interest and reference to where further details about its charter can be found, if not in the protocol. Alternatively, an explanation of why a DMC is not needed	Methods - Ethics Approval and Data and Safety Monitoring
	**28b**	Explanation of any interim analyses and stopping guidelines, including who will have access to these interim results and make the final decision to terminate the trial	Methods - Ethics Approval
Trial monitoring	**29**	Frequency and procedures for monitoring trial conduct. If there is no monitoring, give explanation	Methods - Intervention Delivery and Fidelity
**Ethics**			
Research ethics approval	**30**	Plans for seeking research ethics committee/institutional review board approval	Methods - Ethics Approval
Protocol amendments	**31**	Plans for communicating important protocol modifications to relevant parties	Methods - Ethics Approval
Consent or assent	**32a**	Who will obtain informed consent or assent from potential trial participants or authorized proxies, and how	Methods - Participant Screening; Assessment Visits
	**32b**	Additional consent provisions for collection and use of participant data and biological specimens in ancillary studies, if applicable	Methods - Assessment Visits
Confidentiality	**33**	How personal information about potential and enrolled participants will be collected, shared, and maintained in order to protect confidentiality before, during, and after the trial	Methods - Assessment Visits (REDCap)
Ancillary and post-trial care	**34**	Provisions, if any, for ancillary and post-trial care, and for compensation to those who suffer harm from trial participation	Not applicable
